# Features, Outcome, and Treatment of Postanoxic Status Epilepticus: Pooled Analysis of 3 Cohorts

**DOI:** 10.1212/WNL.0000000000213913

**Published:** 2025-07-25

**Authors:** Pia De Stefano, Jeannette Hofmeijer, Hervé Quintard, Charlotte Damien, Francesco Misirocchi, Sarah Caroyer, Janneke Horn, Selma Tromp, Bert Kornips, Danny Hilkman, Walther van Mook, Cornelia Hoedemaekers, Filippo Annoni, Benjamin Legros, Margitta Seeck, Michel J.A.M. Van Putten, Nicolas Gaspard, for the TELSTAR investigators

**Affiliations:** 1 Neuro-Intensive Care Unit, Department of Intensive Care, University Hospital of Geneva, Switzerland;; 2 EEG & Epilepsy Unit, Department of Clinical Neurosciences, University Hospital of Geneva, Switzerland;; 3 Department of Neurology, Rijnstate Hospital, Arnhem, the Netherlands;; 4 Department of Clinical Neurophysiology, Technical Medical Centre, University of Twente, Enschede, the Netherlands;; 5 Department of Neurology, HUB-Hôpital Erasme, Université Libre de Bruxelles, Belgium;; 6 Unit of Neurology, Department of Medicine and Surgery, University of Parma, Italy;; 7 Amsterdam Coma Group, Amsterdam University Medical Center location Academic Medical Center, the Netherlands;; 8 Department of Neurology and Clinical Neurophysiology, St. Antonius Hospital, Nieuwegein, the Netherlands;; 9 Department of Neurology, Leiden University Medical Center, the Netherlands;; 10 Department of Neurology, VieCuri Medical Center, Venlo, the Netherlands;; 11 Department of Clinical Neurophysiology, Maastricht University Medical Center, the Netherlands;; 12 Department of Intensive Care Medicine, Maastricht University Medical Center & School of Health Professions Education, Maastricht University, the Netherlands;; 13 Department of Intensive Care, Radboud University Medical Center, Nijmegen, the Netherlands;; 14 Department of Intensive Care, Erasme Hospital, Brussels, Belgium;; 15 Departments of Neurology and Clinical Neurophysiology, Medisch Spectrum Twente, Enschede, the Netherlands; and; 16 Department of Neurology, Yale University School of Medicine, New Haven, CT.

## Abstract

**Background and Objectives:**

The prognostic significance and the benefits of antiseizure treatment for definite and possible status epilepticus (SE) after cardiac arrest (CA) remain debated. The study aims to identify clinical and EEG predictors of outcome in definite and possible SE after CA and to determine patient categories in which antiseizure medication is useful.

**Methods:**

We conducted a multicenter pooled analysis of individual patient data from the Treatment of ELectroencephalographic STatus epilepticus After cardiopulmonary Resuscitation trial and 2 local registries (Brussels and Geneva). Patients with EEG patterns fulfilling the American Clinical Neurophysiology Society criteria for definite or possible SE within 72 hours after CA were included. Primary outcome was the cerebral performance category (CPC) at 3 months, dichotomized as good (CPC 1–2) or poor (CPC 3–5). Patients, clinical, EEG, and treatment characteristics were related to outcome using univariate and multivariate analyses in the whole cohort and separate for patients without ≥2 poor outcome European Resuscitation Council (ERC)/European Society of Intensive Care Medicine (ESICM) criteria. This latter group of patients was further divided into 2 subgroups: those with definite SE and those with possible SE.

**Results:**

Of 274 patients (median age 66 [interquartile range (IQR) 55–75], 31% female) with definite or possible SE, 24 (8.8%) had good recovery. In multivariate analysis, nonmotor semiology and SE cessation were associated with good recovery. After exclusion of patients with ≥2 poor outcome ERC/ESICM criteria (180 patients), we included 94 patients (52 definite SE and 42 possible SE), 25% having good outcome. In definite SE, SE cessation (12 [100%] vs 20 [50%], *p* = 0.002), higher discharge frequency (3 Hz [IQR 2–3] vs 2 Hz [IQR 2–3], *p* = 0.024), guideline-recommended SE treatment (12 [100%] vs 28 [70%], *p* = 0.047), and higher doses of levetiracetam (4,250 [IQR 3,750–4,500] mg vs 2,000 [IQR 2,000–3,000] mg, *p* = 0.001) and valproic acid (4,800 [IQR 3,600–5,400] mg vs 2,000 [IQR 1,850–2,250] mg, *p* = 0.032) were associated with favorable outcome. None of the definite or possible SE patients with good outcome had a suppressed/suppression-burst background before SE onset.

**Discussion:**

Patients with postanoxic definite or possible SE have a 25% chance of good outcome in the absence of ≥2 poor outcome ERC/ESICM factors. EEG background continuity before SE onset and higher discharge frequency contribute to the identification of patients who may benefit from protracted treatment.

## Introduction

Epileptiform EEG patterns, including status epilepticus (SE) and other rhythmic and periodic patterns, are reported in 10%–35% of patients with comatose cardiac arrest (CA).^1-3^ These patterns are associated with case fatality rates of 80%–100%.^4,5^ It is unclear whether all epileptiform patterns represent a condition that can be treated with antiseizure medication (ASM) to improve outcome or are merely an epiphenomenon of severe irreversible brain damage in which treatment is futile.^6-9^ Uncertainty about the efficacy of treatment is reflected by debate between experts in the field^6^ and by substantial practice variation.

In 2021, the randomized controlled Treatment of ELectroencephalographic STatus epilepticus After cardiopulmonary Resuscitation (TELSTAR) trial showed no difference in the risk of poor outcome between the intervention and control groups at 3 months after CA.^4,10^ However, for subgroups with patterns fulfilling the American Clinical Neurophysiology Society (ACNS) criteria^11^ for electrographic SE, outcomes in the intervention group were better than in the control group.^10^ The trial was underpowered for these subgroup analyses; however, these results suggest that prolonged antiseizure treatment of patients with postanoxic SE may improve outcome. This is supported by observational^8,9^ and nonrandomized interventional studies.^12^

The TELSTAR trial did not differentiate between definite and possible SE (the latter also referred to as the ictal-interictal continuum) and other rhythmic and periodic discharges falling outside the current consensus criteria for definite or possible SE.^11,13^ The trial did not enroll nor stratify patients based on the severity of brain injury according to guideline-recommended multimodal prognostic tools,^14^ potentially masking treatment effects in patients with less severe postanoxic encephalopathy.^15^ Finally, some control group patients received ASMs or continuous IV sedative medication to suppress visible myoclonus, possibly leading to unintended suppression of definite or possible SE.

With this analysis, we aimed to provide additional insights on the identification of comatose CA patients with definite and possible SE that might have a good outcome and benefit from protracted treatment. The aim of this study therefore was to (1) identify predictors of good outcome and (2) estimate whether treatment with antiseizure and/or sedative medication could be associated with improved outcome.

## Methods

### Study Design

A pooled analysis of individual patient data from 3 cohorts (TELSTAR Trial, Brussels and Geneva Registries) of intensive care unit–admitted comatose patients monitored with continuous EEG (cEEG) or repetitive spot EEG after CA was performed.

### Standard Protocol Approvals, Registrations, and Patient Consents

Studies were approved by local ethic committees (METC Twente NL46296.044.13 and research board of each participating center, Brussels 2014/119 and 2020/483-A2024/082, Geneva CCER 2019-00836). The Strengthening the Reporting of Observational Studies in Epidemiology guidelines were followed to improve the quality of our study. Specificities of study cohorts are detailed in eMethods 1.

### Participants

Comatose patients (Glasgow Coma Scale score ≤8) who had been resuscitated after CA, aged 18 years or older, in whom EEG showed a pattern meeting the ACNS criteria either for definite or for possible SE in the first 72 hours following CA were included in the current analysis.^11^ To this end, all EEGs from the 3 cohorts were re-reviewed by P.D.S. or N.G. blinded for outcome with controversies resolved through consensus meetings. Participants with rhythmic or periodic discharges not meeting either SE or possible SE criteria,^11^ or with unavailable EEG for review, were excluded from this analysis.

### Patient Management

All patients were treated according to national guidelines for post-CA care, that generally followed European guidelines,^14,16^ and SE treatment followed a stepwise approach according to international guidelines.^2,17-19^

Decisions regarding limitation or withdrawal of life-sustaining treatment (WLST) mostly followed the European Resuscitation Council/European Society of Intensive Care Medicine (ERC/ESICM) criteria.^14^

All patients were monitored by cEEG or repetitive spot EEG. eMethods 2 provides detailed information about SE treatment, WLST procedure, and EEG monitoring.

### EEG Analysis

Electrographic SE was defined according to current consensus definitions as epileptiform discharges >2.5 Hz or any pattern with definite spatial or temporal evolution for ≥10 minutes or for a total duration of ≥20% of any 60 minutes period of recording; electroclinical SE was defined if a clinical correlate was time-locked to any rhythmic or periodic EEG pattern or if EEG and clinical improvement were observed after an ASM trial.^11^ Possible electrographic SE, also referred to as the ictal-interictal continuum, is a purely electrographic entity, including any periodic discharge or spike-wave pattern that averages >1 Hz but ≤2.5 Hz over 10 seconds, any periodic discharge or spike-wave pattern that averages ≥0.5 Hz and ≤1 Hz over 10 seconds and has a plus modifier or fluctuation, any lateralized rhythmic delta activity averaging >1 Hz for at least 10 with a plus modifier or fluctuation, and does not qualify as SE.^11^ We also collected the following EEG variables: main terms 1 (localization) and 2 (type of pattern), frequency, evolution, presence of a “plus” modifier,^11^ EEG activity between epileptiform discharges during SE (categorized as visible and present [>10 μV], visible but suppressed [<10 μV], or not visible due to continuous ictal activity) and before SE onset (amplitude, continuity, presence of periodic discharges not fulfilling possible SE criteria, and presence and morphology of bursts), latency to SE, ongoing sedation at time of SE onset, and ongoing targeted temperature management (TTM) at time of SE onset. Continuous background also includes nearly continuous background. In case of multiple different EEG patterns in the same patient, the first pattern meeting the criteria for definite or possible SE was collected and used for analysis. Superrefractory SE was defined as SE that continues or recurs 24 hours or more after the onset of sedative medication.

### Outcomes

The primary outcome measure of this analysis was cerebral performance category (CPC) at 3 months after discharge^20^ for TELSTAR, assessed by a standardized telephone interview by an independent person blinded to treatment allocation, and best CPC within 3 months after discharge for the Brussels and Geneva cohorts, assessed by in-hospital follow-up consultation, dichotomized as good (CPC 1 or 2) and poor (CPC 3–5).

### Other Collected Variables

All data from the TELSTAR database were collected prospectively. Data from the 2 registries were partly collected prospectively and partly supplemented with additional information retrospectively collected from EEG reviews and electronic medical records (eTable 1). Collected variables include

- Demographics (age, sex).
- CA features (time to return of spontaneous circulation [ROSC], cardiac vs noncardiac etiology, and initial shockable vs nonshockable rhythm).
- Early management features (sedation during the first 24 hours, TTM at 33–34°C).
- Treatment variables after detection of definite or possible SE (administration of first-line benzodiazepine, type and maximal daily dose of ASMs, type and maximal infusion rate of sedative medication, refractoriness, and superrefractoriness).^21^ All participants who received sedative drug and ASM doses meeting the recommendations^2^ were considered as “guideline recommended treated.”

Prognostic criteria for unfavorable prognosis were largely based on the ERC/ESICM criteria^14^ (eMethods 3) but modified as follows: we did not exclude patients with myoclonus, even in case of possible status myoclonus because we did not want to exclude patients with potentially treatable myoclonic SE. Second, the EEG criterion (presence of a highly malignant EEG) was applied before possible SE onset and thus sometimes before 24 hours, although European guidelines recommend considering EEG data after 24 hours.^14^ This decision was based on the observation that some SE patterns, such as evolving generalized periodic discharges (GPDs) on a suppressed background, may already meet the criterion at SE onset, and we aimed to avoid confusion between background EEG and definite or possible SE patterns.

### Statistical Analyses

We performed a first analysis at the whole group level, including all patients with definite or possible SE. Next, we excluded patients with ≥2 ERC/ERCM criteria for unfavorable prognosis. The remaining group of patients was further divided into 2 subgroups: those with definite SE and those with possible SE. Variables are presented as count (frequency) or median (interquartile range [IQR]), as appropriate. Univariate comparisons between patients with good and poor outcome were performed using the χ^2^ or Fisher exact test for categorical variables and the Mann-Whitney or Student *t* test for continuous variables, depending on normality assessed by the Kolmogorov-Smirnov test. Multivariate analysis was used to identify factors independently associated with outcome. Given the complete separation observed in the distribution of some variables between groups (observed count of 0 in 1 or more groups), penalized logistic regression with Firth correction was used to mitigate the risk of small sample bias. The small size of the definite and possible SE groups precluded any multivariate analysis. A *p* value <0.05 was considered statistically significant. Statistical analyses were conducted using JASP (version 0.18.3; JASP Team 2024) and R (version 4.1.2; R Core Team 2021).

### Data Availability

The data sets used and/or analyzed during this study are available from the corresponding author on reasonable request.

## Results

### Patients' Demographics and Clinical Features

**Figure 1:**
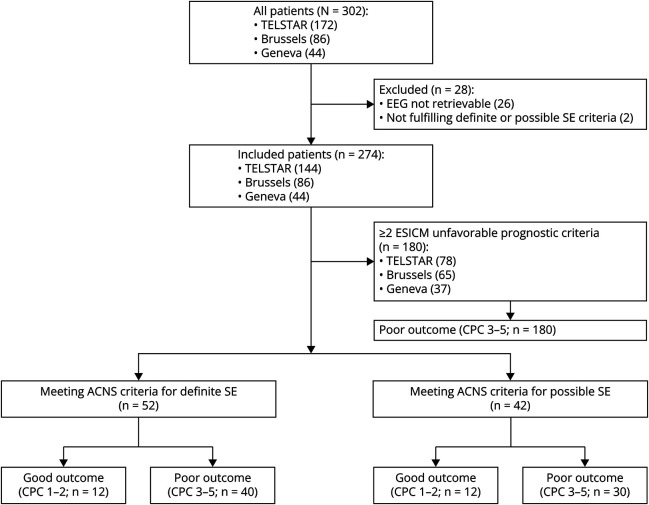
Patients' Flowchart ACNS = American Clinical Neurophysiology Society; CPC = cerebral performance category; ESICM = European Society of Intensive Care Medicine; SE = status epilepticus; TELSTAR = Treatment of ELectroencephalographic STatus epilepticus After cardiopulmonary Resuscitation Trial.

**Figure 2:**
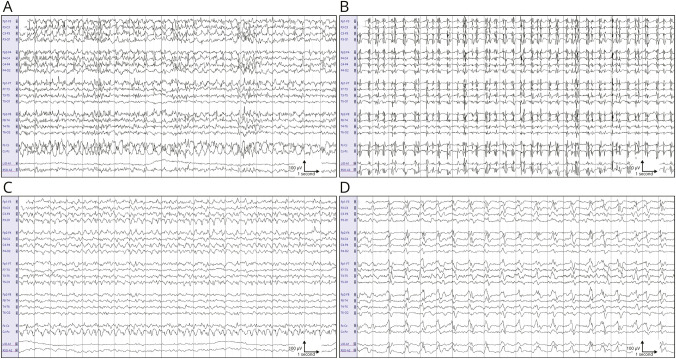
EEG Examples of Definite and Possible Status Epilepticus With Good and Poor Outcome (A) Definite status epilepticus of a patient with good outcome. (B) Definite status epilepticus of a patient with poor outcome. (C) Possible status epilepticus of a patient with good outcome. (D) Possible status epilepticus of a patient with poor outcome. All examples are 20 seconds epochs, and the EEG is displayed in a bipolar longitudinal montage. Filter setting: high-pass 0.53 Hz, low-pass 70 Hz.

A total of 302 participants was included in the 3 cohorts (Figure 1). Of those, 28 were excluded (26 because no EEG was available for review and 2 because they did not meet definite or possible SE criteria), leaving a total of 274 participants for analysis. The median age was 66 (IQR 55–75) years, with 31% being female. The majority (89%) suffered from out-of-hospital CA, and 52% had a shockable rhythm. The cause of arrest was primary cardiac in 69% of cases. The median time to ROSC was 20 (IQR 15–30) minutes, and only 36% of patients underwent TTM at 33–34°C. Sedation during the first 24 hours was administered to 98% of patients. Median time from hospital admission to EEG recording was 15 (IQR 11–23) hours, and median latency of definite or possible SE from admission was 32 (IQR 21–40) hours, with 53% of patients having definite SE with prominent motor symptoms, 29% having definite nonconvulsive SE, and 28% showing possible SE (Figure 2). Periodic discharges were present in the majority (57%) of patients, 31% showed ictal discharges, 9% spike-waves, and 3% rhythmic delta activity.

### Outcomes and Prognostic Factors in the Whole Group of Patients

Twenty-four (8.8%) patients had a good neurologic outcome (Table 1). Sex, out-of-hospital CA, shockable rhythm, cardiac etiology, time to ROSC, and TTM were not associated with outcome. Latency to definite SE or possible SE onset was significantly longer in patients with good outcome (median 44 hours [IQR 20–42] vs 32 hours [IQR 34–53], *p* < 0.001), with SE with prominent motor symptoms associated with poor outcome (*p* = 0.010). No single ACNS main term was associated with outcome, except for the presence of a suppressed activity between epileptiform discharges during SE (*p* = 0.007). SE cessation was associated with good outcome (*p* < 0.001), while superrefractory SE and the presence of ≥2 ERC/ESICM criteria for unfavorable prognosis were related to poor outcome (*p* = 0.038 and *p* < 0.001, respectively). Specifically, no patient with a “highly malignant” EEG background in the first 24 hours or before definite or possible SE onset had a good outcome. In addition, of those who had a good outcome, all had either a continuous (18 [75%]) or discontinuous EEG background (6 [25%]), and none had a suppressed or suppression-burst background before definite or possible SE onset (*p* = 0.001). In multivariate analysis, the presence of ≥2 ERC/ESICM unfavorable prognostic criteria (odds ratio [OR] 0.00, 95% CI 0.00–0.01, *p* < 0.001) and SE with motor features (OR 0.49, 95% CI 0.12–0.87, *p* = 0.025) were negatively associated with good outcome, while SE cessation (OR 2.23, 95% CI 1.26–3.28, *p* = 0.006) was positively associated with good outcome. None of the patients presenting a GPD on a suppressed background survived.

**Table 1:** Patient Characteristics of the Whole Cohort and According to Outcome

	All (N = 274)	CPC 3–5 (N = 250)	CPC 1–2 (N = 24)	*p* Value (univariate)	*p* Value (multivariate)	Odds ratio (multivariate)
Age, y	66 (55–75)	63 (54–70)	66 (56–75)	0.160		
Sex, female	84 (31)	75 (30)	9 (38)	0.596		
Out-of-hospital arrest	244 (89)	222 (89)	22 (92)	>0.999		
Shockable rhythm	143 (52)	126 (50)	17 (71)	0.157		
Cardiac etiology	188 (69)	168 (67)	20 (83)	0.114		
Time to ROSC (N = 231), min	20 (15–30)	20 (15–30)	15 (15–27)	0.324		
TTM to 33–34°C	98 (36)	91 (36)	7 (29)	0.656		
Sedation during the first 24 h	268 (98)	244 (98)	24 (100)	>0.999		
Latency to definite/possible SE, h	32 (21–40)	32 (20–42)	44 (34–53)	<0.001	0.652	1.00 (0.99–1.02)
Sedation at definite/possible SE onset	214 (78)	195 (78)	19 (79)	>0.999		
TTM at definite/possible SE onset	52 (19)	50 (20)	2 (8)	0.273		
Definite/possible SE classification				0.010	0.025 (SE with motor features vs NCSE/possible SE)	0.49 (0.12–0.87) (SE with motor features vs NCSE/possible SE)
SE with motor features	145 (53)	139 (56)	6 (25)			
NCSE	52 (29)	46 (18)	6 (25)			
Possible SE	77 (28)	65 (26)	12 (50)			
ACNS main term 1				0.478		
Generalized	267 (97)	244 (98)	23 (96)			
Lateralized	6 (2)	5 (2)	1 (4)			
Bilateral independent	1 (0)	1 (0)	0 (0)			
ACNS main term 2				0.582		
Periodic discharges	157 (57)	145 (58)	12 (50)			
Rhythmic delta activity	7 (3)	6 (2)	1 (4)			
Spike-and-wave	26 (9)	23 (9)	3 (13)			
Ictal discharge^a^	84 (31)	76 (30)	8 (33)			
Frequency, c/s	2 (2–3)	2 (2–3)	2 (2–3)	0.239		
Static	172 (63)	157 (63)	15 (63)	0.077		
Fluctuating	55 (20)	47 (19)	8 (33)			
Evolving	47 (17)	46 (18)	1 (4)			
Plus modifier	74 (27)	67 (27)	7 (29)	0.993		
EEG background between discharges during SE				0.007	0.383 (visible and suppressed vs others)	0.87 (0.29–1.60) (visible and suppressed vs others)
Suppressed	97 (35)	95 (38)	2 (8)			
Not suppressed	91 (33)	80 (32)	11 (46)			
Not assessable	86 (31)	75 (30)	11 (46)			
Guideline-recommended SE treatment	217 (79)	196 (78)	21 (88)	0.432		
Refractory SE	189 (69)	173 (56)	16 (67)	0.998		
Superrefractory SE	89 (32)	86 (34)	3 (13)	0.038	0.116	0.64 (0.10–1.29)
Cessation of SE	120 (44)	100 (40)	20 (83)	<0.001	0.006	2.23 (1.26–3.28)
≥2 ERC/ESICM criteria for unfavorable prognosis	180 (66)	180 (72)	0 (0)	<0.001	<0.001	0.00 (0.00–0.01)
EEG background before SE (N = 264)				<0.001	Not included	Not included
Highly malignant	100 (38)	100 (42)	0 (0)			
Discontinuous	83 (31)	77 (32)	6 (25)			
Continuous and nearly continuous	81 (30)	63 (26)	18 (75)			

Abbreviations: ACNS = American Clinical Neurophysiology Society; CPC = cerebral performance category; ERC = European Resuscitation Council; (E)SE = (electrographic) status epilepticus; ESICM = European Society of Intensive Care Medicine; NCSE = nonconvulsive status epilepticus; ROSC = return of spontaneous circulation; TTM = targeted temperature management.

Data are presented as count (percentage) or median (interquartile range).

a Ictal discharges include rhythmic or periodic epileptiform discharges ≥3 Hz, rhythmic discharges >4 Hz or any evolving discharge ≥0.5 Hz. Effective treatment = if patients received doses of antiseizure and sedative medication in line with SE treatment recommendations^2^; superrefractory SE = if persistent after a first cycle (≥24 hours) of sedative medication; SE cessation = resolution of rhythmic and periodic patterns fulfilling definite or possible SE definitions.^11,13^.

### Outcome and Prognostic Factors After Exclusion of Patients With ≥2 Unfavorable Outcome Predictors

After excluding patients with ≥2 unfavorable outcome predictors based on ESICM/ERC criteria, 94 patients presenting either definite SE (52) or possible SE (42) were analyzed. These are described consecutively below.

### Both Definite and Possible SE

Characteristics of patients are summarized in Table 2. Patients with good outcome (24 [25%]) had similar demographics, CA, and management features compared with those with poor outcome. Latency to SE onset and SE semiology did not differ between the 2 groups. Regarding EEG features, higher discharge frequency was associated with good outcome (2.5 Hz [IQR 2–3] vs 2 Hz [IQR 1.5–2.5], *p* = 0.014). Of those with a good outcome, 18 (67%) and 6 (33%) had a continuous or discontinuous background before SE onset, respectively. None of the good outcome patients had a suppressed or suppression-burst background before SE onset. Higher doses of levetiracetam (3,000 [IQR 3,000–4,500] mg vs 2,000 [IQR 2,000–3,000] mg, *p* = 0.005) and valproic acid (4,400 [IQR 3,000–5,000] mg vs 2,000 [IQR 1,900–3,000] mg, *p* = 0.015) and SE cessation were associated with good outcome (*p* = 0.008). In multivariate analysis, only SE cessation remained associated with good outcome (OR 3.92, 95% CI 1.20–13.63, *p* = 0.010).

**Table 2:** Features of All Patients (Definite and Possible SE) With <2 Unfavorable Outcome Predictors

	All (N = 94)	CPC 3–5 (N = 70)	CPC 1–2 (N = 24)	*p* Value (univariate)	*p* Value (multivariate)	Odds ratio (multivariate)
Age, y	63 (56–74)	63 (56–75)	63 (54–70)	0.388		
Sex, female	32 (34)	23 (33)	9 (38)	0.679		
Out-of-hospital arrest	84 (89)	62 (89)	22 (92)	>0.999		
Shockable rhythm	66 (70)	49 (70)	17 (71)	>0.999		
Cardiac etiology	79 (84)	59 (84)	20 (83)	>0.999		
Time to ROSC (N = 86), min	17 (15–25)	19 (14–25)	15 (15–25)	0.633		
TTM to 33–34°C	24 (25)	17 (24)	7 (29)	0.873		
Sedation during the first 24 h	92 (98)	68 (97)	24 (100)	>0.999		
Latency to definite SE, h	40 (32–51)	40 (31–47)	44 (34–53)	0.199		
Sedation at definite SE onset	76 (81)	57 (81)	19 (79)	>0.999		
TTM at definite SE onset	8 (9)	6 (9)	2 (8)	>0.999		
SE classification				0.388		
Definite SE with motor features	34 (36)	28 (40)	6 (25)			
Definite NCSE	18 (19)	12 (17)	6 (25)			
Possible SE	42 (45)	30 (43)	12 (50)			
ACNS main term 1				>0.999		
Generalized	91 (97)	68 (97)	23 (96)			
Lateralized	3 (3)	2 (3)	1 (4)			
ACNS main term 2				0.158		
Periodic discharges	62 (66)	50 (71)	12 (50)			
Rhythmic delta activity	5 (5)	4 (6)	1 (4)			
Spike-and-wave	8 (9)	5 (7)	3 (13)			
Ictal discharge^a^	19 (20)	11 (16)	8 (33)			
Frequency (Hz)	2 (1.5–2.5)	2 (1.5–2.5)	2.5 (2.0–3.0)	0.014	0.181	1.48 (0.86–2.98)
Static	58 (62)	43 (61)	15 (63)	0.675		
Fluctuating	27 (29)	19 (27)	8 (33)			
Evolving	9 (10)	8 (11)	1 (4)			
Plus modifier	33 (35)	26 (37)	7 (29)	0.480		
EEG background before SE (N = 92)				0.050		
Highly malignant	2 (2)	2 (3)	0 (0)			
Discontinuous	40 (43)	34 (49)	6 (33)			
Continuous and nearly continuous	50 (55)	32 (46)	18 (67)			
EEG background between discharges during SE				0.278		
Suppressed	4 (4)	2 (3)	2 (8)			
Not suppressed	52 (55)	41 (59)	11 (46)			
Not assessable	38 (40)	27 (39)	11 (46)			
Guideline-recommended SE treatment	67 (71)	46 (66)	21 (88)	0.056		
Refractory SE	56 (60)	40 (57)	16 (67)	0.562		
Superrefractory SE	17 (18)	14 (20)	3 (13)	0.546		
Cessation of SE	56 (60)	36 (55)	20 (100)	0.008	0.010	3.92 (1.20–13.63)
First-line benzodiazepine bolus	16 (17)	11 (16)	5 (21)	0.543		
IV ASMs						
Levetiracetam	49 (52)	36 (51)	13 (54)	0.817		
Maximum daily dose, mg	2,000 (2,000–3,000)	2,000 (2,000–3,000)	3,000 (3,000–4,500)	0.006	0.133	1.01 (1.00–1.02)
Valproate	33 (35)	23 (33)	10 (42)	0.435		
Maximum daily dose, mg	2,500 (2,000–4,200)	2,000 (1,900–3,000)	4,400 (3,000–5,000)	0.015	0.214	1.01 (1.00–1.02)
Lacosamide	13 (14)	9 (13)	4 (17)	0.641		
Maximum daily dose, mg	400 (200–400)	300 (200–400)	400 (400–500)	0.098		
Phenytoin	16 (17)	14 (20)	2 (8)	0.228		
Maximum daily dose, mg	1,500 (1,350–1,800)	1,500 (1,300–1,700)	1,750 (1,600–1,900)	0.333		
Continuous IV sedative medication						
Midazolam	36 (38)	26 (37)	10 (42)	0.694		
Maximum infusion rate, mg/h	10 (5–17)	8 (5–16)	12 (10–18)	0.241		
Propofol	50 (53)	38 (54)	12 (50)	0.717		
Maximum infusion rate, mg/h	230 (172–300)	240 (200–315)	204 (38–240)	0.167		
Thiopental	2 (2)	2 (3)	0 (0)	>0.999		
Maximum infusion rate, mg/h	800	800	NaN	NaN		
Ketamine	1 (1)	1 ()	0 (0)	>0.999		
Maximum infusion rate, mg/h	200	200	NaN	NaN		

Abbreviations: ACNS = American Clinical Neurophysiology Society; ASM = antiseizure medication; CPC = cerebral performance category; NCSE = nonconvulsive status epilepticus; ROSC = return of spontaneous circulation; SE = status epilepticus; TTM = targeted temperature management.

Data are presented as count (percentage) or median (interquartile range).

a Ictal discharges include rhythmic or periodic epileptiform discharges ≥3 Hz, rhythmic discharges >4 Hz or any evolving discharge ≥0.5 Hz.

### Definite SE (Both Electroclinical and Electrographic SE)

Characteristics of patients with SE are summarized in Table 3. Patients with good outcome (12 [23%]) had similar demographics, CA, and management features compared with those with poor outcome. Latency to SE onset and SE semiology did not differ between the 2 groups. In 3 cases, SE started within 24 hours after arrest, including 1 with a latency to onset of 20 hours and good outcome. Regarding EEG features, higher discharge frequency was associated with good outcome (3 Hz [IQR 2–3] vs 2 Hz [IQR 2–3], *p* = 0.024). This was irrespective of the ACNS main terms 1 and 2 and EEG background pattern during SE. Of those with a good outcome, 8 (67%) and 4 (33%) had a continuous or discontinuous background before SE onset, respectively. None of the good outcome patients had a suppressed or suppression-burst background before SE onset.

**Table 3:** Features of the Group of Patients With Favorable Prognostic Indicators and Definite SE

	All (N = 52)	CPC 3–5 (N = 40)	CPC 1–2 (N = 12)	*p* Value (univariate)
Age, y	62 (56–73)	62 (56–74)	63 (58–70)	0.888
Sex, female	20 (38)	14 (35)	6 (50)	0.550
Out-of-hospital arrest	49 (94)	37 (93)	12 (100)	>0.999
Shockable rhythm	34 (65)	26 (65)	8 (67)	>0.999
Cardiac etiology	43 (83)	34 (85)	9 (75)	0.415
Time to ROSC (N = 44), min	19 (15–25)	20 (15–25)	15 (15–27)	0.633
TTM to 33–34°C	11 (21)	7 (18)	4 (33)	0.253
Sedation during the first 24 h	50 (96)	38 (95)	12 (100)	>0.999
Latency to definite SE, h	40 (32–50)	40 (32–47)	46 (35–53)	0.415
Sedation at definite SE onset	43 (83)	33 (83)	10 (83)	>0.999
TTM at definite SE onset	5 (10)	3 (8)	2 (17)	0.325
Definite SE classification				0.352
Definite SE with motor features	34 (65)	28 (70)	6 (50)	
NCSE	18 (35)	12 (30)	6 (50)	
ACNS main term 1				0.553
Generalized	49 (94)	38 (95)	11 (92)	
Lateralized	3 (6)	2 (5)	1 (8)	
ACNS main term 2				0.071
Periodic discharges	27 (52)	24 (60)	3 (25)	
Rhythmic delta activity	1 (2)	1 (3)	0 (0)	
Spike-and-wave	5 (10)	4 (10)	1 (8)	
Ictal discharge^a^	19 (37)	11 (28)	8 (67)	
Frequency, Hz	3 (2–3)	2 (2–3)	3 (2–3)	0.024
Static	30 (58)	21 (53)	9 (75)	0.475
Fluctuating	13 (25)	11 (28)	2 (17)	
Evolving	9 (17)	8 (20)	1 (8)	
Plus modifier	19 (37)	15 (38)	4 (33)	>0.999
EEG background before SE (N = 51)				0.638
Highly malignant	1 (2)	1 (3)	0 (0)	
Discontinuous	21 (40)	17 (43)	4 (33)	
Continuous and nearly continuous	29 (56)	21 (53)	8 (67)	
EEG background between discharges during definite SE				0.172
Suppressed	2 (4)	1 (3)	1 (8)	
Not suppressed	24 (46)	21 (53)	3 (25)	
Not assessable	26 (50)	18 (45)	8 (67)	
Guideline-recommended SE treatment	40 (79)	28 (70)	12 (100)	0.047
Refractory SE	36 (63)	25 (63)	11 (92)	0.118
Superrefractory definite SE	9 (17)	7 (18)	2 (17)	>0.999
Cessation of definite SE	32 (62)	20 (50)	12 (100)	0.002
First-line benzodiazepine bolus	14 (27)	9 (23)	5 (42)	0.267
IV ASMs				
Levetiracetam	33 (63)	25 (63)	8 (67)	>0.999
Maximum daily dose, mg	2,000 (2,000–3,500)	2,000 (2,000–3,000)	4,250 (3,750–4,500)	0.001
Valproate	22 (42)	15 (38)	7 (58)	0.318
Maximum daily dose, mg	2,000 (2,000–4,725)	2,000 (1,850–2,250)	4,800 (3,600–5,400)	0.032
Lacosamide	10 (19)	7 (18)	3 (25)	0.679
Maximum daily dose, mg	400 (200–400)	200 (200–400)	400 (400–500)	0.167
Phenytoin	6 (12)	6 (15)	0 (0)	0.316
Maximum daily dose, mg	1,500 (1,425–1,500)	1,500 (1,425–1,500)	NaN	NaN
Continuous IV sedative medication				
Midazolam	23 (44)	17 (43)	6 (50)	0.746
Maximum infusion rate, mg/h	14 (8–19)	12 (6–20)	15 (11–18)	0.673
Propofol	28 (54)	21 (53)	7 (58)	0.754
Maximum infusion rate, mg/h	225 (168–305)	245 (180–320)	208 (122–225)	0.253
Thiopental	1 (2)	1 (3)	0 (0)	>0.999
Maximum infusion rate, mg/h	800	800	NaN	NaN
Ketamine	1 (2)	1 (3)	0 (0)	>0.999
Maximum infusion rate, mg/h	200	200	NaN	NaN

Abbreviations: ACNS = American Clinical Neurophysiology Society; ASM = antiseizure medication; CPC = cerebral performance category; NCSE = nonconvulsive status epilepticus; ROSC = return of spontaneous circulation; SE = status epilepticus; TTM = targeted temperature management.

Data are presented as count (percentage) or median (interquartile range).

a Ictal discharges include rhythmic or periodic epileptiform discharges ≥3 Hz, rhythmic discharges >4 Hz or any evolving discharge ≥0.5 Hz.

Guideline-recommended SE treatment and SE cessation were associated with good outcome (*p* = 0.047 and *p* = 0.004, respectively). Although no specific ASM or sedative medication demonstrated superiority in chance of a good outcome and doses of levetiracetam (4,250 [IQR 3,750–4,500] mg vs 2,000 [IQR 2,000–3,000] mg, *p* = 0.001) and valproic acid (4,800 [IQR 3,600–5,400] mg vs 2,000 [IQR 1,850–2,250] mg, *p* = 0.032) were higher in patients with good than in those with a poor outcome. Similarly, doses of lacosamide (400 [IQR 400–500] mg vs 200 [IQR 200–400] mg) were higher in patients with a good outcome, but this difference did not reach statistical significance (*p* = 0.167). There was no difference in the number of medications used between poor and good outcomes (2 median, *p* = 0.616).

### Possible (Electrographic) SE

Characteristics of patients with possible SE are summarized in Table 4. Twelve (29%) patients had good outcome. They had similar demographics and CA features and management variables compared with those with poor outcome. Latency to possible SE onset and EEG features were similar between the 2 groups. No difference was found in treatment type and doses, either. Finally, cessation of possible SE (*p* = 0.002), but not guideline-recommended SE treatment, was associated with better outcome. Of those with a good outcome, 10 (83%) and 2 (17%) had a continuous or discontinuous background before SE onset, respectively. None of the good outcome patients had a suppressed or suppression-burst background before SE onset. There was no difference in the number of medications used between poor and good outcomes (1 median, *p* = 1.000)

**Table 4:** Features of the Group of Patients With Favorable Prognostic Indicators and Possible SE

	All (N = 42)	CPC 3–5 (N = 30)	CPC 1–2 (N = 12)	*p* Value (univariate)
Age, y	65 (55–75)	66 (57–77)	62 (53–68)	0.259
Sex, female	12 (29)	9 (30)	3 (25)	>0.999
Out-of-hospital arrest	35 (83)	25 (83)	10 (83)	>0.999
Shockable rhythm	32 (76)	23 (77)	9 (75)	>0.999
Cardiac etiology	36 (86)	25 (83)	11 (92)	0.655
Time to ROSC (N = 42), min	15 (11–26)	15 (12–25)	18 (11–27)	0.949
TTM to 33–34°C	13 (31)	10 (33)	3 (25)	0.723
Sedation during the first 24 h	42 (100)	30 (100)	12 (100)	>0.999
Latency to possible SE, h	40 (32–51)	38 (29–47)	44 (34–58)	0.303
Sedation at possible SE onset	33 (79)	24 (80)	9 (75)	0.699
TTM at possible SE onset	3 (7)	3 (10)	0 (0)	0.545
ACNS main term 1: Generalized	42 (100)	30 (100)	12 (100)	>0.999
ACNS main term 2				0.322
Periodic discharges	35 (83)	26 (87)	9 (75)	
Rhythmic delta activity	4 (10)	3 (10)	1 (8)	
Spike-and-wave	3 (7)	1 (3)	2 (17)	
Frequency, Hz	1.7 (1.4–2.0)	1.5 (1.3–2.0)	2.0 (1.6–2.0)	0.222
Static	28 (67)	22 (73)	6 (50)	0.169
Fluctuating	14 (33)	8 (27)	6 (50)	
Plus modifier	14 (33)	11 (37)	3 (25)	0.719
EEG background before SE (N = 41)				0.021
Highly malignant	1 (2)	1 (3)	0 (0)	
Discontinuous	19 (45)	17 (57)	2 (17)	
Continuous and nearly continuous	21 (50)	11 (37)	10 (83)	
EEG background between discharges during possible SE				0.724
Visible and suppressed	2 (5)	1 (3)	1 (8)	
Visible and nonsuppressed	28 (67)	20 (67)	8 (67)	
Not visible because of possible SE	12 (29)	9 (30)	3 (25)	
Guideline-recommended SE treatment	27 (64)	18 (60)	9 (75)	0.485
Refractory SE	20 (48)	15 (50)	5 (42)	0.884
Superrefractory possible SE	8 (19)	7 (23)	1 (8)	0.402
Cessation of possible SE	24 (57)	14 (47)	11 (92)	0.002
First-line benzodiazepine bolus	2 (5)	2 (7)	0 (0)	>0.999
IV ASMs				
Levetiracetam	16 (38)	11 (37)	5 (42)	>0.999
Maximum daily dose, mg	2,500 (2,000–3,000)	2,000 (2,000–3,000)	3,000 (2,000–3,500)	0.926
Valproate	11 (26)	8 (27)	3 (25)	>0.999
Maximum daily dose, mg	3,000 (2,000–3,900)	2,400 (1,900–3,200)	3,800 (3,100–4,000)	0.364
Lacosamide	3 (7)	2 (6)	1 (8)	>0.999
Maximum daily dose, mg	400 (300–400)	350 (325–350)	400	NaN
Phenytoin	10 (24)	8 (27)	2 (17)	0.696
Maximum daily dose, mg	1,500 (1,300–1,900)	1,500 (1,200–1,800)	1,750 (1,600–1,900)	>0.999
Continuous IV sedative medication				
Midazolam	13 (31)	9 (30)	4 (33)	>0.999
Maximum infusion rate, mg/h	5 (5–8)	5 (3–8)	8 (5–20)	0.235
Propofol	22 (52)	17 (57)	5 (42)	0.499
Maximum infusion rate, mg/h	230 (197–263)	232 (200–300)	200 (100–240)	0.237
Thiopental	1 (2)	1 (3)	0 (0)	>0.999
Maximum infusion rate, mg/h	970	970	NaN	NaN

Abbreviations: ACNS = American Clinical Neurophysiology Society; ASM = antiseizure medication; CPC = cerebral performance category; NCSE = nonconvulsive status epilepticus; ROSC = return of spontaneous circulation; SE = status epilepticus; TTM = targeted temperature management.

Data are presented as count (percentage) or median (interquartile range).

## Discussion

In this pooled analysis, we found that definite or possible SE does not preclude a good outcome, with an overall 9% patients having CPC 1–2 at 3 months and even higher proportions after excluding patients with ≥2 poor outcome factors according to ERC/ESICM guidelines,^14^ reaching 25% for the whole cohort, 23% for definite SE, and 29% for possible SE. Shorter latency from arrest to definite or possible SE onset, the presence of motor manifestations, and EEG background suppression were associated with a poor outcome. However, none of these features in isolation irrevocably predicted poor outcome.

Although suppressed EEG activity between discharges and the presence of motor manifestations were associated with poor outcome in univariate analysis, none of these predicted poor outcome without false positives. Furthermore, only the latter was independently associated with poor outcome in multivariate analysis, and none were associated with outcome in the SE subgroups, in line with recent evidence.^22^ After excluding patients with ≥2 poor outcome factors according to ERC/ESICM guidelines, higher frequency of discharges was related to good outcome in the whole group and in patients with definite SE with a median frequency of 2.5 Hz and 3 Hz, respectively, both fulfilling the EEG criteria of SE,^11^ whereas no EEG features or treatment variables were related to good or poor outcome in patients with possible SE. This indicates that outcome prediction based on EEG and clinical features is challenging in patients with possible SE.

The temporal evolution of EEG background features, particularly a return to a continuous or discontinuous background before the emergence of rhythmic and periodic discharges, was a significant prognostic factor in a post hoc analysis of the TELSTAR trial, and in line with previous findings.^23,24^ We found that the return to a continuous or discontinuous background before SE onset, as opposed to SE emerging directly from a suppressed or a suppression-burst background, was a necessary condition for a good outcome. This, and the variable and sometimes short (<24 hours) latency period from arrest to SE onset, stresses the need for early EEG monitoring in patients with CA for optimal prognostication and SE management.^25^

For the whole group of patients with definite or possible SE, cessation of SE was associated with good outcome, while administration of guideline-recommended SE treatment was not. One possible explanation includes the relatively small sample size of the groups investigated. However, there are some other plausible explanations for this apparent paradox. Either postanoxic SE is a self-resolving phenomenon in patients with a good outcome, which thus requires no treatment, either of the current treatment protocols is not sufficiently effective or aggressive for this condition, leading to good response in some patients but not in others, or postanoxic SE is refractory in patients with poor outcome, making any treatment futile. All these explanations remain possible and not mutually exclusive. Aggravation of short-term synaptic depression and potentiation of excitatory neurotransmission presumably play a key role in irreversible brain injury.^26^ Further dedicated prospective comparative studies are needed to further clarify the prognostic and physiologic significance of the various EEG patterns. Imaging and serum biomarkers might possibly provide additional insights.

Furthermore, in the subgroup of patients with definite SE and <2 ERC/ESICM predictors of unfavorable outcome, the use of antiseizure and sedative medication according to guidelines was associated with good outcome.

Although no specific antiseizure or sedative drug demonstrated superiority, higher doses of ASM (particularly levetiracetam and valproic acid) were associated with better outcome in the entire cohort. However, this association remained significant only in the definite SE group, supporting the evidence that patients with definite SE may benefit from treatment, whereas patients with possible SE may not, a condition that is likely to be self-resolving.

Superrefractoriness^21^ was associated with unfavorable outcomes in the entire cohort in univariate analysis. However, this association was no longer significant after adjusting for potential confounders, including the suspected severity of brain injury, indicating that, in itself, SE superrefractoriness is not a sign of irreversible brain injury. Similarly, in the subgroups of patients with definite or possible SE and <2 ERC/ESICM predictors of unfavorable outcome, the association was no longer significant.

Favorable outcomes were reported in more than 40% of post-CA survivors with refractory SE (defined as failure of benzodiazepines and a first IV ASMs) and preserved SSEPs following aggressive and prolonged treatment.^12^ This study and our findings suggest that, in selected patients with postanoxic SE fulfilling consensus criteria^11,13^ and no evidence of severe irreversible anoxic brain injury,^14^ aggressive and prolonged therapy might lead to a good outcome.

Strengths of our study include the multicentric design and standardized collection of EEG, clinical, and outcome data, the use of the most recent and validated EEG terminology on definite and possible SE and guidelines^11,13,27^ and the analysis of subgroups of patients with <2 poor outcome factors following ERC/ESICM guidelines.^14^ All EEGs were reanalyzed by experienced EEG readers. However, several limitations should be considered. First, as in all observational studies, the nonrandomized nature hampers strong conclusions about effects of treatment. For example, choices for higher dosages of medication or longer treatment may have been related to patients' characteristics and clinical context, thus introducing allocation bias. Second, 7% of patients were not monitored with cEEG, increasing the risk of missing epileptiform pattern onset, especially if nonconvulsive. Third, although we had access to treatment details such as type and dose, additional information such as the precise temporal course of each drug might have helped shedding more light on possible relations between treatment characteristics and outcome. Finally, the small sample size, particularly for the definite and possible SE subcategories, warrants caution in interpreting results.

The present analysis of data from 274 individual patients from 3 databases confirms that EEG patterns meeting criteria for definite or possible SE are not necessarily related to a poor outcome in comatose CA survivors: the probability of a good neurologic outcome at 3 months was 25% (23%–29%) in the absence of ≥2 poor outcome factors according to ERC/ESICM guideline.^14^ Patients with a good outcome always had return to a continuous or discontinuous EEG background pattern before the onset of definite or possible SE, a higher discharge frequency and received higher ASMs doses, particularly the patients with definite SE. Clinical semiology (motor vs nonconvulsive) did not influence prognosis. The association between guideline-recommended treatment and good neurologic outcome suggests that selected comatose CA patients with definite SE may benefit from protracted intensive treatment and care. However, the evidence to recommend specific treatments remains insufficient. To establish clear treatment guidelines, further randomized controlled trials are needed.

## Appendix Coinvestigators

**Table:**

Coinvestigators are listed at Neurology.org.

## Glossary

ACNS: American Clinical Neurophysiology Society
ASM: antiseizure medication
CA: cardiac arrest
cEEG: continuous EEG
CPC: cerebral performance category
ERC: European Resuscitation Council
ESICM: European Society of Intensive Care Medicine
GPD: generalized periodic discharge
IQR: interquartile range
OR: odds ratio
ROSC: return of spontaneous circulation
SE: status epilepticus
TELSTAR: Treatment of ELectroencephalographic STatus epilepticus After cardiopulmonary Resuscitation
TTM: targeted temperature management
WLST: withdrawal of life-sustaining treatment
